# Changes in Cell Membrane Fatty Acid Composition of *Streptococcus thermophilus* in Response to Gradually Increasing Heat Temperature

**DOI:** 10.4014/jmb.1912.12053

**Published:** 2020-03-02

**Authors:** Bonggyu Min, Kkotnim Kim, Vladimir Li, Seoae Cho, Heebal Kim

**Affiliations:** 1 Department of Agricultural Biotechnology and Research Institute of Agriculture and Life Sciences, Seoul National University, Seoul 08826, Republic of Korea; 2 Interdisciplinary Program in Bioinformatics, Seoul National University, Seoul 0886, Republic of Korea; 3 C&K genomics Inc., C-1008, H businesspark, Seoul 08826, Republic of Korea

**Keywords:** Probiotics, *Streptococcus thermophilus*, heat Adaptation, cross protection, fatty acid composition, membrane fluidity

## Abstract

In this study, a method of heat adaptation was implemented in an attempt to increase the upper thermal threshold of two *Streptococcus thermophilus* found in South Korea and identified the alterations in membrane fatty acid composition to adaptive response to heat. In order to develop heat tolerant lactic acid bacteria, heat treatment was continuously applied to bacteria by increasing temperature from 60°C until the point that no surviving cell was detected. Our results indicated significant increase in heat tolerance of heat-adapted strains compared to the wild type (WT) strains. In particular, the survival ratio of basically low heat-tolerant strain increased even more. In addition, the strains with improved heat tolerance acquired cross protection, which improved their survival ratio in acid, bile salts and osmotic conditions. A relation between heat tolerance and membrane fatty acid composition was identified. As a result of heat adaptation, the ratio of unsaturated to saturated fatty acids (UFA/SFA) and C18:1 relative concentration were decreased. C6:0 in only heatadapted strains and C22:0 in only the naturally high heat tolerant strain were detected. These results support the hypothesis, that the consequent increase of SFA ratio is a cellular response to environmental stresses such as high temperatures, and it is able to protect the cells from acid, bile salts and osmotic conditions via cross protection. This study demonstrated that the increase in heat tolerance can be utilized as a mean to improve bacterial tolerance against various environmental stresses.

## Introduction

Probiotics are living microorganisms whose inclusion in the diet intends to provide health benefits of the host by improving intestinal environment [1]. Lactic acid bacteria (LAB) belong to a class of probiotic bacteria that has been extensively used in food industries and especially dairy product industry. *Streptococcus thermophilus* belongs to LAB class bacteria, and it has been extensively used in the food industry [2]. As suggested by its name, *S. thermophilus* is thermophilic lactic acid bacteria. They have the ability to withstand relatively high temperatures. During past decades, this species has been commonly used for the production of fermented dairy foods because it both enhances the flavor of dairy products, and accelerates the ratio of product acidification [3].

During industrial processes, bacteria may experience various environmental stresses such as low pH and osmotic pressure. Heat stress has been one of the most widely studied topics of LAB [4]. Elevated temperatures (> 60°C) are required for certain manufacturing processes such as pasteurization, which can negatively affect bacterial growth and even lead to cell death [5]. This led to the increased interests in expanding LAB abilities to survive under these heat stress conditions, several studies on their heat tolerance were reported [6, 7].

Stress adaptation among LAB varies with respect to bacterial species and stress conditions, but the critical response to the adaptation of LABs at various stresses involves major changes in membrane fatty acid composition [8]. Several studies have shown that a link between the membrane fatty acid composition and the bacterial heat tolerance has been found that the adaptation of bacteria in the stress conditions induced change of cell membrane fatty acid composition [9, 10]. The ratio between unsaturated and saturated fatty acids (UFA/SFA) is inversely correlated with the growth temperature [11]. Zhang and Rock stated that bacterial cells can control the biosynthesis of new fatty acids or can modify the structure of existing ones, and this allows bacteria to alter cell membrane fluidity and rapidly adapt to the changed environmental conditions [12].

The aim of this study was to identify the adaptive response to heat and the modifications in membrane fatty acid composition of *S. thermophilus* after its exposure to high temperature. Previous studies have been carried out about resistance to temperature related stresses of *S. thermophilus* [13, 14]. However, to our knowledge, there is no study available for *S. thermophilus* that the responses of cells after exposed heat stress over 60 degrees or a relation of adaptation of this stressful condition and the changes of membrane fatty acid composition. In our study, we applied a newly designed adaptive evolutionary method to induce heat adaptation of *S. thermophilus* strains. The heat tolerance of two *S. thermophilus*, BIOPOP-1 and BIOPOP-2, isolated from fermented dairy foods in South Korea was improved as adaptation in gradually increasing heat temperature condition. In addition, the difference in improve stress tolerance of these two strains that inherently high heat tolerance and those having low heat tolerance when cultured at 50°C was identified. This study provided not only an approach to improve bacterial tolerance response in high temperature but also an insight into the stress cross protection [15]. The alteration in cell membrane fatty acid composition of *S. thermophilus* strains achieved by adaptation through only heat-shock response in high temperature without additional stress. It was hypothesized that the heat-adapted bacteria not only tolerate high temperature, but also can withstand various stress conditions. Through this study, we proved that one cause of these reasons is alteration of cell membrane fatty acid composition. This method can be applied in wide range of industries.

## Materials and Methods

### Isolation and Selection of Heat Tolerant Bacterial Strains

Several strains were isolated from fermented dairy foods in South Korea and only 8 catalase negative and Gram-positive isolates were selected [16]. They were cultured in sterile deMan Rogosa Sharpe medium (MRS, Difco, Becton Dickinson Co., USA) and incubated at 37°C for 24 h. To screen for naturally heat tolerant strains, cells were incubated at 50°C for 24 h and two surviving isolates were selected. The cells were labeled as BIOPOP-1 and BIOPOP-2 and stored as stock samples in 40% glycerol at -80°C [17].

### Identification of the Isolates with 16S rRNA Gene Sequencing

BIOPOP-1 and BIOPOP-2 strains were identified using 16S ribosomal RNA (rRNA) gene sequencing method. Genomic DNA was extracted according to the instruction provided by the manufacturer of DNA extraction kit (QIAGEN, USA) [16]. The 16S rRNA gene was amplified using the universal bacterial primer sets: 27F 5' (AGA GTT TGA TCM TGG CTC AG) 3' and 1492R 5' (TAC GGY TAC CTT GTT ACG ACT T) 3' [18]. Amplified PCR products were sent for sequencing (Macrogen, Korea) and then results were used for assigning taxonomy using EZ-Biocloud server [19]. The phylogenetic trees were built based on the 16S rRNA gene sequences using the neighbor-joining methods by the MEGA-X software [20]. The 16S rRNA gene sequences of 12 *Streptococci* strains and one *Lactococcus lactis* for using as out group were downloaded from the National Center for Biotechnology Information (NCBI) database.

### Heat Adaptation Procedure

Two strains of *S. thermophilus*, BIOPOP-1 and BIOPOP-2, were the starting material for the experiment [4]. Cultures from the stocks were streaked on the MRS agar plate and incubated at 37°C for 48 h. Each colony was isolated and transferred to 10 ml MRS and incubated at 37°C for 24 h. After incubation, 10 μl of each sample was transferred to 1.5 ml micro tube with 990 μl MRS broth pre-heated at test temperature and heat treatment was performed in a dry bath for 1 min. Samples were cooled down for 5 min at room temperature, and incubated at 37°C for 24 h. This procedure was repeated two more times and then the temperature was increased by 3°C. Repeated incremental heat treatment was performed starting from 60°C until the point all bacteria were not detected. Heat-treated strains, which underwent the procedure for three days, were collected after incubation to measure changes in their heat tolerance levels at each temperature point. [21]. Collected samples then were stored at −80°C in 40% glycerol as stock solutions and the final surviving strains were designated as heat-adapted strains.

### Identification of Heat Tolerance Enhancement

The stocks of each temperature sample collected in the above step were thawed at room temperature and streaked on MRS agar plates. They were incubated at 37°C for 48 h and then each single colony was individually transferred to tubes with 10 ml MRS broth and incubated at 37°C for 24 h. 10 μl of each sample was transferred to 1.5 ml micro tube with 990 μl MRS broth pre-heated at 72°C and heated for 1 min using a dry bath. After the heat treatment, they were cooled down for about 5 min at room temperature. Samples were serially diluted with 0.85%saline, then spread on MRS agar plates and incubated at 37°C for 48 h. The survival ratio was calculated by dividing Colony-Forming Units (CFUs) of the stress treated cultures by the CFU of non-treated (control) cultures [22].

### Viability Comparison between WT and Heat-Adapted Strains

**Heat treatment with variable temperatures at the set time.** The stocks of WT and heat-adapted strains were thawed at room temperature and streaked on agar plates. After incubated the plates at 37°C for 48 h, isolated single colonies of each plate were transferred into test tubes with 10 ml of MRS and incubated at 37°C for 24 h. Heat treatment was performed in dry bath with base temperature set to 60°C for both WT and heat-adapted strains, temperature increment was 3°C until final survival temperature was reached for each sample. Cells were inoculated in MRS broth (10 μ cells, 990 μl media) at each temperature point from 60 to final survived temperature of each strains and heated for 1 min. They were then serially diluted with 0.85% saline and transferred on MRS agar plate and incubated at 37°C for 48 h.

**Prolonged heat treatment at constant temperature.** Subcultures of WT and heat-adapted strains were prepared using samples from previous part. They inoculated 100 ul into 10 ml pre-heated MRS broth and heated in a water bath at 60°C from 0 to 50 min 100 μl of the cells were transferred to tubes with 10 ml pre-heated MRS broth [23]. The survival ratio was checked every 10 min. After heat treatment, they were left to cool down for 5 min at room temperature. Cells then were serially diluted with 0.85% saline and spread on MRS agar plates and incubated at 37°C for 48 h.

The D-value (decimal reduction time) was determined given by the equation:

t = D × (log N_0_ − logN_f_),

where t: time (min), D: D-value at heat conditions, N_0_: initial concentration of microorganisms, N_f_: final concentration of microorganisms [24]. D-value of each strain was calculated as the negative inverses of the regression line slopes obtained by plotting the log number of survivors against time [25].

### Assessment of Tolerance Enhancement in Other Stresses after Heat Adaptation

The stocks of WT and heat-adapted strains were thawed at room temperature and streaked on agar plates. After incubated the plates at 37°C for 48 h, isolated single colonies of each plate were transferred into test tubes with 10 ml of MRS and incubated at 37 °C for 24 h. Cells were then harvested by centrifugation (4,000 rpm, 10 min, and 4°C). They were washed twice with phosphate-buffered saline (PBS) with pH 7.0. To measure response against acid, cell pellets were re-suspended with MRS broth adjusted to 2, 3, and 7 (control) [26]. Cell suspensions were incubated at 37°C for 2 h. To evaluate their viability, they were serially diluted and spread on MRS agar plates, then incubated at 37°C for 48 h.

Bile salt tolerance of each strain was examined. Cells were harvested following the same protocol as in the acid tolerance experiment and re-suspended by MRS containing 0.5% and 1% bile salts (cholic acid sodium salt 50%and deoxycholic acid sodium salt 50%, Sigma Aldrich, 48305) [26]. Cell suspensions were incubated at 37°C for 3 h. Then serially diluted, spread on MRS agar plates and incubated at 37°C for 48 h.

To assess osmotic tolerance, bacteria were harvested following the same protocol as in the acid tolerance experiment. Cell pellets were then re-suspended by MRS containing 20% NaCl (Sodium chloride, 99.5%). The cell suspensions were incubated at 37°C for 2 h and 24 h, serially diluted, spread on MRS agar plates. Then, plates incubated at 37°C for 48 h. The survival ratio was calculated by dividing CFUs of the stress treated cultures by the CFU of non-treated (control) cultures [22].

### Analysis of Fatty Acid Component of Bacterial Membrane

Fatty acids analysis was performed according to the method outlined by Garces and Mancha [27]. The stocks of WT and heat-adapted strains were thawed at room temperature and streaked on agar plates. Plates were then incubated at 37°C for 48 h, single colonies from each plate were transferred into test tubes with 10 ml of MRS and incubated at 37°C for 24 h. Cells were then harvested by centrifugation and washed twice with distilled water. Pellets were transferred to tubes with Teflon-lined caps and pentadecenoic acid (15:0) was used as an internal standard. Samples were mixed with methylation mixture containing methanol, benzene, DMP (2, 2-Dimethoxy-propane), sulfuric acid (H_2_SO_4_) and heptane. For lipid extraction tubes were placed in a water bath at 80°C for 2 h. They were then cooled down at room temperature. The samples were shaken, and The samples were shaken, and left to settle, after which the content formed two layers. The top layer containing Fatty Acid Methyl Esters (FAMEs) was extracted and analyzed using Agilent 7890A gas chromatography (Agilent, USA) equipped with a flame ionization detector (FID) and a DB-23 column (60 mm × 0.25 mm × 0.25 um) (Agilent Technologies, Inc., Wilmington, DE). GC settings: injector temperature 250°C, split ratio 10:1, carrier flow 1.2 ml/min, detector temperature 280°C, air flow in detector 350 ml/min, hydrogen flow 35 ml/min. The results were shown as relative percentages of each fatty acid and the ratios of saturated fatty acids (SFAs) and unsaturated fatty acids (UFAs) were calculated [23].

### Statistical Analysis

All experiments were conducted three times. The Colony-Forming Units (CFUs) were counted and the viability was calculated by dividing the CFUs of the test cultures by the CFUs of non-treated (control) [22]. The results were indicated as mean ± SD (standard deviation) [26]. Independent t-tests for statistical analyses were performed using R software [28] and *P*-value was considered statistically significant (*p* < 0.05) [16].

## Results

### Screening and Phylogenetic Analysis of the Strains

Strains were isolated from fermented dairy foods and appeared as gram-positive and catalase-negative bacteria. Two globular-shaped strains BIOPOP-1 and BIOPOP-2 were selected after incubation at 50°C for 24 h. Strain BIOPOP-1 demonstrated adequate survival and proliferation ratios (97.59 ± 1.40%), while strain BIOPOP-2 showed positive survival ratio, comparatively low proliferation ratio (7.06 ± 0.67%).

**Fig. 1:**
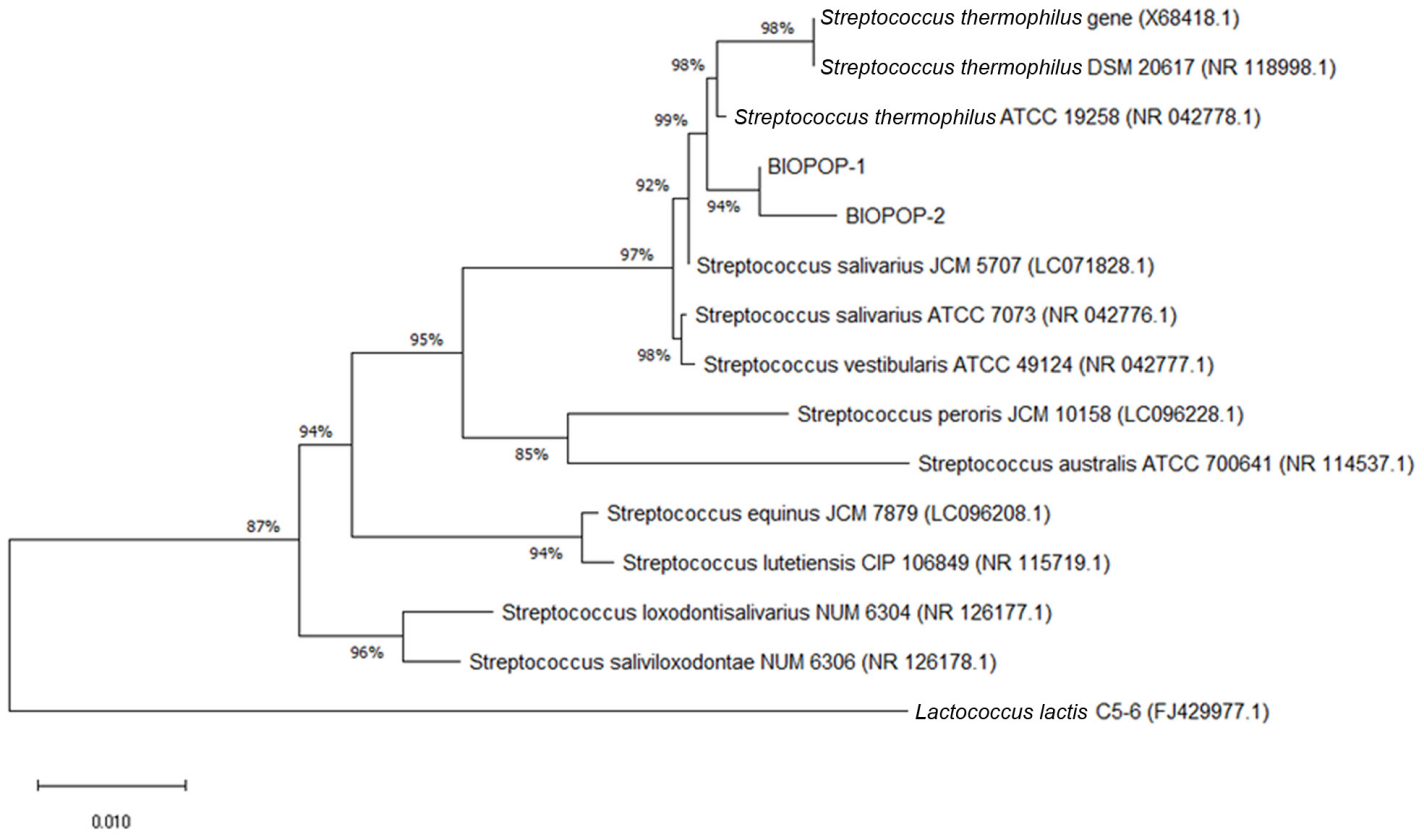
Phylogenetic relationship of the isolates with related taxa based on 16S rRNA sequences. Neighbourjoining tree showing the phylogenetic relationships of strain BIOPOP-1, strain BIOPOP-2 and related type strains. 16S rRNA gene sequence of *Lactococcus lactis* was used as out group.

Phylogenetic tree based on the 16S rRNA gene sequences was built (Fig. 1). According to it, strains BIOPOP-1 and BIOPOP-2 were identified as *Streptococcus thermophilus*. The 16S rRNA gene of strain BIOPOP-1 had 99.8%match with *S. thermophilus* type strain ATCC 19258 and strain BIOPOP-2’s 16S rRNA gene had 99.25% match with *S. thermophilus* type strain ATCC 19258 (Table S1) [29]. Although strains BIOPOP-1 and BIOPOP-2 were of the same species, they showed completely different levels of natural heat tolerance.

### Increasing Bacterial Heat Tolerance Threshold by Heat Adaptation

**Fig. 2:**
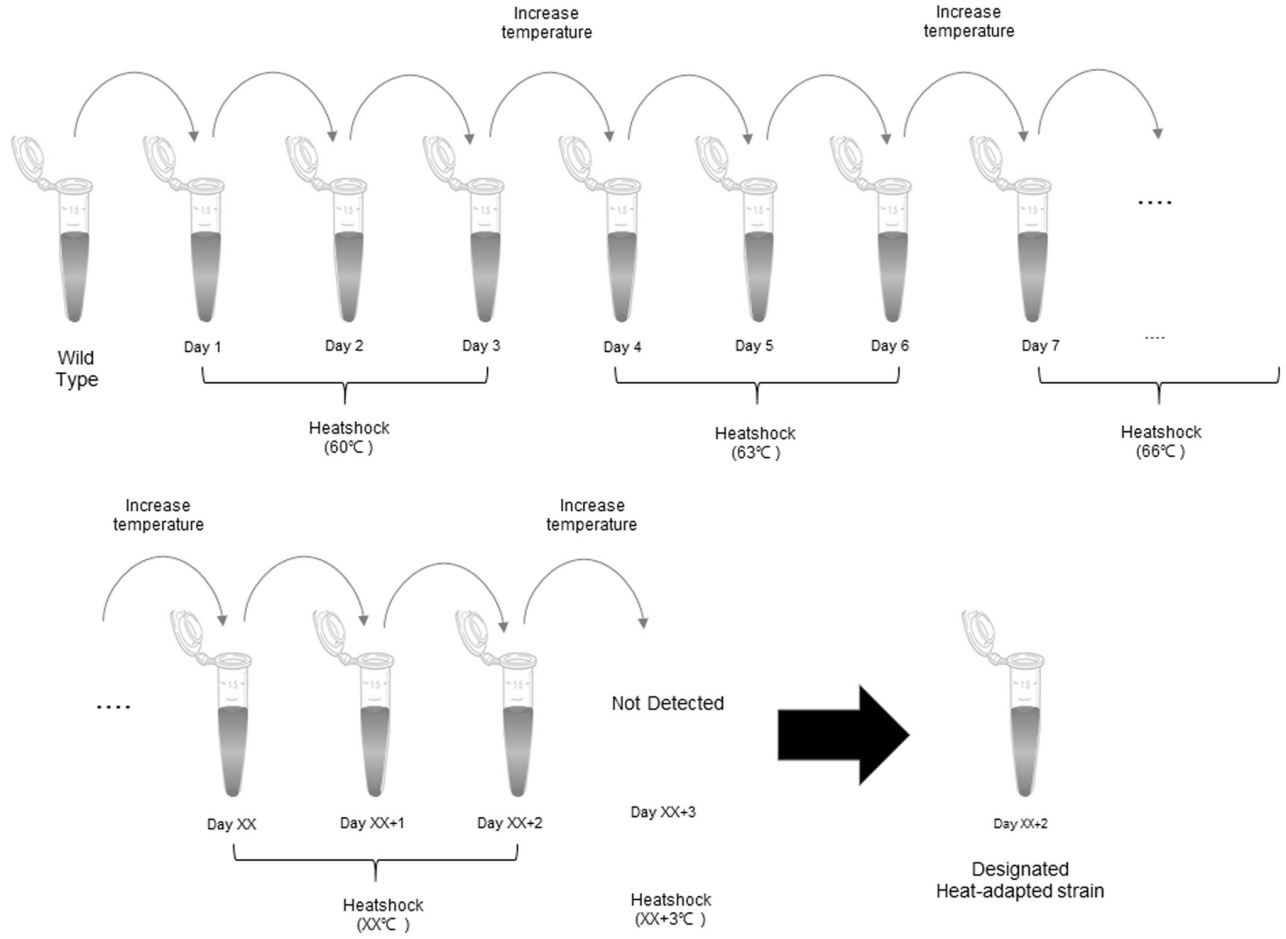
Procedure for heat adaptation experiment. Temperatures was gradually increased from 60°C until strains were not detected. 10 μl of each sample was transferred to 1.5 ml micro tube with 990 μl MRS broth pre-heated at test temperature. Heat shock time was 1 min and then incubated at 37°C for 24 h. This procedure was repeated three times and increased temperature (3°C). The final surviving bacteria were designated as heat-adapted strains.

The cells were subjected to heat adaptation procedure by gradually elevating the base (60°C) temperature [30]. The process of this experiment is outlined in Fig. 2. BIOPOP-1 strain was able to withstand temperatures up to 84°C, while BIOPOP-2 strain only survived up until 81°C was reached. The surviving bacteria were designated as heat-adapted strains (BIOPOP-1: 84°C, BIOPOP-2: 81°C).

**Fig. 3:**
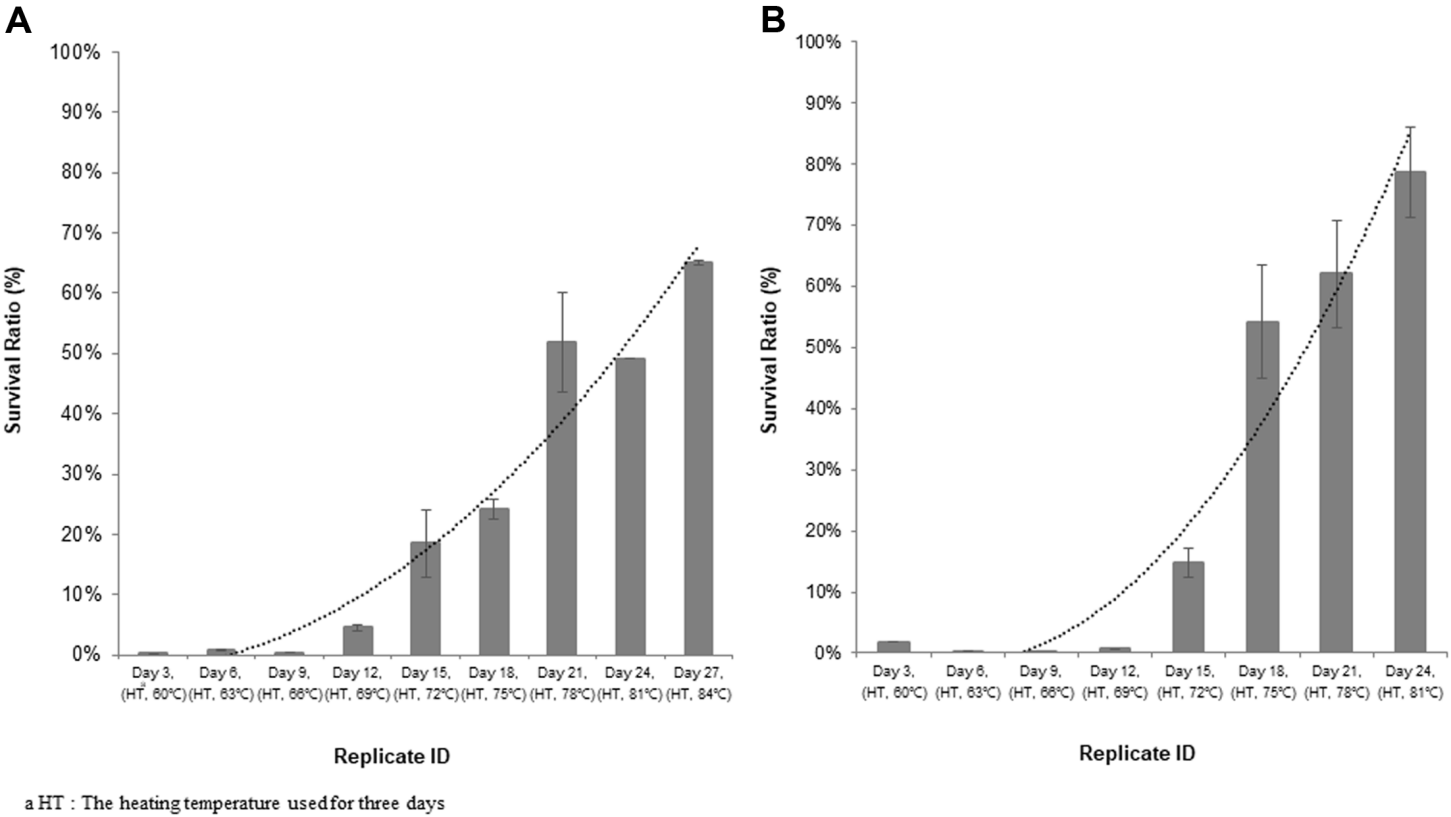
Enhancement of heat tolerance threshold of *S. thermophilus*. (**A**) *S. thermophilus* BIOPOP-1, (**B**) *S. thermophilus* BIOPOP-2. X axis represents strains that were taken from the last step (day 3) of each heat treatment temperature in heat-adaptation process. HT means the heating temperature used for three days and Y axis presents the percentage of strain’s survival ratio (%) that was calculated by dividing the CFUs of the heat-treated cultures by the CFUs of non-treated (control). The error bars represent the calculated standard deviation of the measurements of three biological replicates.

To assess enhanced heat tolerance of the strains, we thawed the stocks that were made the third day of each temperature during heat-adaptation procedure. They were cultured and subjected to the heat shock at 72°C for 1 min. Heat shock temperature, 72°C, is a midpoint within acceptable temperature range for the both strains, and is a deciding criterion for using in the test. As a result, BIOPOP-1 strain demonstrated that 60°C strain was the lowest viability and the low viability was almost maintained up to 66°C strain. However, the viability was gradually increased from 69°C strain and 84°C (heat-adapted) strain was the highest (Fig. 3A). In case of BIOPOP-2 strain, cell viabilities were low up to 69°C strains, but it drastically increased from 72°C strain. The highest viability data were recorded 81°C (heat-adapted) strain (Fig. 3B).

### Viability Comparison between WT and Heat-Adapted Strains

**Fig. 4:**
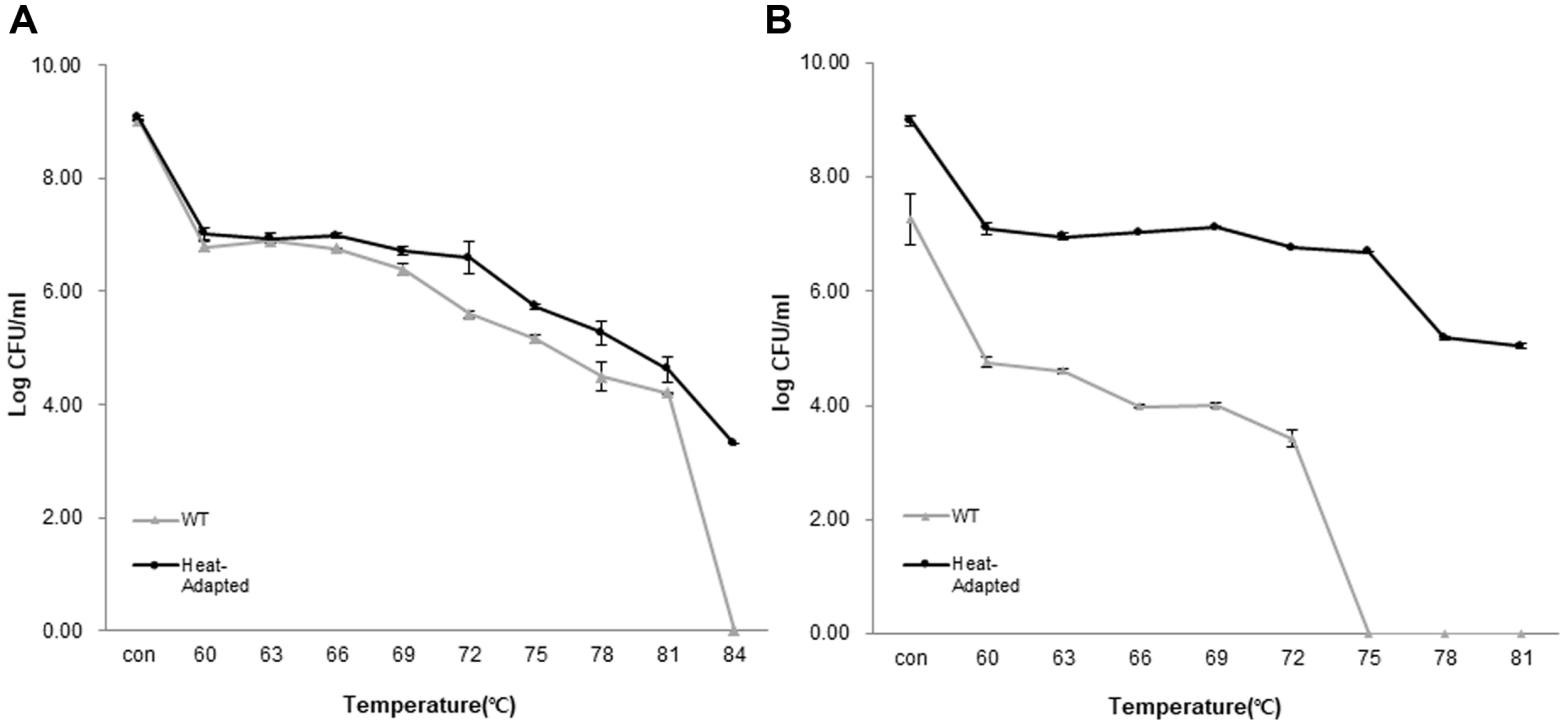
The results of heat treatment with variable temperatures at the set time. (**A**) *S. thermophilus* BIOPOP-1, (**B**) *S. thermophilus* BIOPOP-2. Heat shock time was set at 1 min and heated from 60°C to strains final temperature. The survival ratio of the bacteria was determined by counting the CFUs on MRS agar plate and expressed in log values. The error bars represent the calculated standard deviation of the measurements of three biological replicates.

**Heat shock with variable temperatures at set time.** Starting from basal 60°C, cells were subjected to heat treatment with gradual temperature increment of 3°C every 1 min until final survival temperature was reached; 84°C for BIOPOP-1 and 81°C for BIOPOP-2 correspondingly. BIOPOP-1 strain showed no significant difference in cell viability between WT and heat-adapted strains. Nevertheless, the viability of heat-adapted strain was slightly higher than the WT sample. Upon reaching 84°C, no viable WT cells were observed, while most of the heat-adapted strain cells remained alive (Fig. 4A). In case of BIOPOP-2 strain, WT cells were not detected from temperature reached 75°C, while the heat-adapted strain were detected until reaching 81°C (Fig. 4B). Overall, the results demonstrated positive increment of heat tolerance in heat-adapted strains compared to WT.

**Fig. 5:**
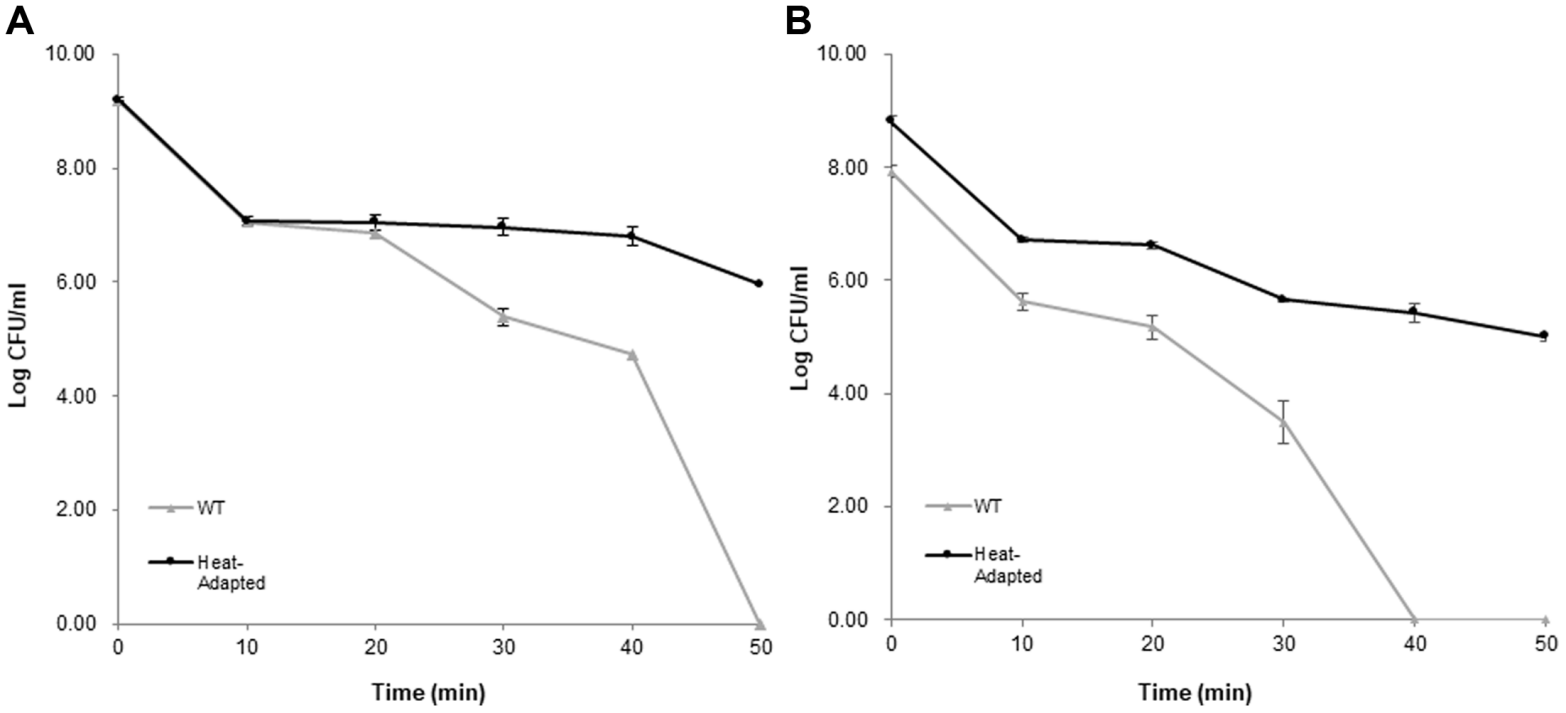
The results of prolonged heat treatment at constant temperature. (**A**) *S. thermophilus* BIOPOP-1, (**B**) *S. thermophilus* BIOPOP-2. The heat temperature was set to 60°C and the heat treatment proceeded until 50 min. Survival ratio was checked every 10 min. The survival ratio of the bacteria was determined by counting the CFUs on MRS agar plate and expressed in log values. The error bars represent the calculated standard deviation of the measurements of three biological replicates.

**Prolonged heat treatment at constant temperature.** Cells were heated at 60°C for 0 to 50 min to assess their heat tolerance level [23]. The cell viability of heat-adapted strain was much higher than that of WT cells for the most of the time. In case of strain BIOPOP-1, the results of WT strain and heat-adapted strain were similar until reaching 10 min time period. However, the number of viable cells started to gradually different after 20 min, and no growth was detected at 50 min time limit, but heat-adapted strain was still within the adequate values (Fig. 5A). Likewise, the viability of BIOPOP-2 showed that no growth of WT cells was detected after 40 min time period, while heat-adapted strain remained alive until 50 min time limit (Fig. 5B).

Microorganisms defined their heat tolerance by D-value (decimal reduction time) which is exposure time required to causes one log10 or 90% reduction of the initial population under specified temperature [18]. Comparing WT and heat-adapted strains, heat-adapted strain of BIOPOP-1 was higher (D-value of 2.0 min) than WT strain (D-value of 1.4 min) and BIOPOP-2 heat-adapted strain was also higher than WT strain that D-value of WT strain was 1.1 min and heat-adapted strain was 2.7 min [25]. The result proved that heat-adapted strains enhanced their heat tolerance through heat adaptation procedure and the overall results of this method positively correlate with the results of the upper method, demonstrating stable positive increment towards heat survivability of heat-adapted strains.

### Heat Adaptation Induced Cross Protection Enhancement against Various Environmental Stresses

The higher the heat tolerance, the stronger tolerance to other stresses by cross protection [31]. In order to confirm this, the strains with increased heat tolerance through heat-adapted experiment were exposed to various stress environments such as acid, bile salt and salinity. Table 1 summarizes the results of cross protection in these stress conditions.

**Acid tolerance.** Acid tolerance is one of the vital abilities of LABs necessary to survive in acidic environment of the stomach after being ingested [32]. The average pH of the stomach is 2~3 and it takes about 2~3 h for food to move through the digestive tract [33]. WT and heat-adapted strains were exposed deMan Rogosa sharpe (MRS) medium where the pH was adjusted to 2 and 3. When exposed to pH 3 forll strains showed that survival ratios were similarly high. The WT of BIOPOP-1 strain was 78.18% and WT of BIOPOP-2 was 69.81%, while that of BIOPOP-1’s heat-adapted strain was 83.13% and strain BIOPOP-2 was 91.76%. However, exposing bacteria to pH 2 for 2 h showed significant change in survival ratios that all strains were dramatically decreased than exposing bacteria to pH 3. The survival ratio was that WT strain of both BIOPOP-1 and BIOPOP-2 was 0.002%, while that of BIOPOP-1 heat-adapted strain was 0.48% and BIOPOP-2 was 0.53%.

**Bile salts tolerance.** The ability of lactic acid bacteria to survive under bile salts environments is also important for probiotics [26]. Both WT and heat-adapted strains were exposed to 0.5% bile salts for consequent 3 h. The survival ratio of BIOPOP-1 (WT) was 1.75% and BIOPOP-2 (WT) was 0.19%, while that of BIOPOP-1 (heat-adapted) was 71.82% and BIOPOP-2 (heat-adapted) was 98.91%. Cells were then exposed to 1.0% bile salts for 3 h, and while survival ratio of BIOPOP-1 (heat-adapted) was 0.29% and BIOPOP-2 (heat-adapted) was 0.28%; WT cells were not detected at all. Thus, the results revealed the heat-adapted strains also acquired improved bile tolerance induced by heat.

**Osmotic tolerance.** One of the methods usually used for preserving foods is to increase osmotic pressure by supplementing salts such as NaCl or KCl [34]. To evaluate osmotic tolerance, WT and heat-adapted strains were first exposed to MRS containing 10% NaCl for 24 h, and both strains demonstrated 100% survival ratio. When exposed to MRS containing 20% NaCl for 2 h, the survival ratio of BIOPOP-1 (WT) dropped to 10.88% and BIOPOP-2 (WT) to 5.35%, while that of BIOPOP-1 (heat-adapted) was 47.62% and BIOPOP-2 (heat-adapted) was 29.34%. Lastly, the bacteria were exposed to 20% NaCl for 24 h, and demonstrated the following survival ratios: BIOPOP-1 (WT): 0.29%, BIOPOP-2 (WT): 0.16%, and BIOPOP-1 (heat-adapted): 6.63%, BIOPOP-2 (heat-adapted): 7.88%. Again, these data positively correlate with the previous stress experiments

### Influence of Heat Adaptation on Membrane Fatty Acid Composition of *S. thermophilus*

A chromatographic method was used to determine the membrane fatty acid composition of *S. thermophilus* WT and heat-adapted strains [11]. The fatty acid compositions of *S. thermophilus* BIOPOP-1 and BIOPOP-2 strains are summarized details in Table 2. *S. thermophilus* BIOPOP-1 WT strain’s membrane is made of seven fatty acids and that of heat-adapted strain consists of eight fatty acids. Also, in case of BIOPOP-2, membranes of WT and heat-adapted strains are composed of six and seven fatty acids correspondingly. The six common peaks in all strains were as follows; hexadecanoic (palmitic) acid (C16:0), octadecanoic (stearic) acid (C18:0), oleic acids (C18:1), arachidic (icosanoic) acid (C20:0), eicosenoic acid (C20:1), and erucic acids (C22:1) [25]. The results between the WT and heat-adapted strains were observed that the compositions of hexanoic acids (C6:0) and oleic acids (C18:1) were different. C18:1 composition of BIOPOP-1 and BIOPOP-2 heat-adapted strains were lower (14.99 % and 21.61%, respectively) than that of WT strains (18.7 % and 25.97%). Also, C6:0 was not detected in BIOPOP-1 and BIOPOP-2 WT strains, but heat-adapted strains were detected (BIOPOP-1: 0.34%, BIOPOP-2: 0.35%). In addition, when comparing the BIOPOP-1 and BIOPOP-2 strains, only BIOPOP-1 strains was confirmed to have behenic (docosanoic) acid (C22:0): 0.34% for WT strain and 1.19% for heat-adapted strain.

The total fatty acid compositions were divided into two groups: saturated fatty acids (SFAs) and unsaturated fatty acids (UFAs) [23]. When we compared fatty acid composition of WT and heat-adapted strains, the total SFAs concentration of BIOPOP-1 heat-adapted strain was slightly higher that of WT strain, 42.09% and 42.52%correspondingly. In contrast, the total UFAs concentration decreased 57.91% for WT cells and 57.48% for heat-adapted strain. In case of BIOPOP-2, the total SFAs concentration of heat-adapted strain was higher (34.37%) than that of WT strain (25.06%). On the contrary, the total UFAs concentration of heat-adapted was 65.63% and it was less than WT (74.94%). As a result, both BIOPOP-1 and BIOPOP-2 featured increased ratios for saturated fatty acids, and reduced for unsaturated fatty acids. The UFA/SFA ratio are commonly used as indirect indicators of membrane fluidity [11]. The previous study was reported that high UFA/SFA ratio show a high membrane fluidity [35]. The UFA/SFA ratio of BIOPOP-1 and BIOPOP-2 observed for heat-adapted cells were lower (1.35 and 1.91, respectively) than WT (1.38 and 2.99, respectively). By analyzing these results, it can be observed with the decreased ratio between UFA/SFA, tolerance to various stresses increases [13].

## Discussion

Heat tolerance is one of the most important abilities of LABs necessary to survive during manufacturing processes, such as food fermentation or pasteurization, in which they can be exposed to high temperatures (up to 60°C) [5]. One study showed that heat tolerant *Escherichia coli* were developed by continuously cultivating the bacteria at 48.5°C [36]. In another study, researchers were able to increase the survival temperature of *Corynebacterium glutamicum* from 33°C to 41.5°C [37]. However, very few studies about manipulating tolerance of *S. thermophilus* strains through stress adaptation exist, and no studies have been done dedicated to manipulating the bacteria’s heat tolerance in over 60°C.

In this study, bacterial strains with elevated heat tolerance threshold were developed using heat adaptation method as shown Fig. 2. All experiments were carried out under sterile conditions and the risk of contamination during the experiments were eliminated. Several probiotic strains primarily isolated from fermented dairy foods in South Korea and two *S. thermophilus* of LAB that were able to survive at 50°C for 24 h were selected for this study. These two strains showed the difference that inherently high heat tolerance (BIOPOP-1) and those having low heat tolerance (BIOPOP-2) when cultured at 50°C. The growth of strain BIOPOP-1 was able to proliferate well (97.59 ± 1.40%), while BIOPOP-2 survived but hardly grew (7.06 ± 0.67%). This explains that strains of the same species can have different thresholds of heat tolerance. The adaptive evolution method was applied to the bacteria by gradually increasing the temperature and the final surviving bacteria were designated heat-adapted strains.

Fig. 3 shows that detectable changes in both strains started 72°C strains, and increased until achieving 84°C for BIOPOP-1, and 81°C for BIOPOP-2. Significant difference in the readings observed between start (60°C) and each end (BIOPOP-1: 84°C, BIOPOP-2: 81°C) strains, suggesting that bacteria increased heat tolerance to a greater extent. It is theorized that the evolutionary shifts of both strains were triggered around temperature points over 70°C.

Two types of heat treatment experiments to compare viability between WT and heat-adapted strains conducted and the overall results matched with the hypothesis that the viabilities of heat-adapted strains were relatively higher than those of WT strains (Figs. 4 and 5). Also, WT strains were completely absent during the final stage of each experiment, whereas heat-adapted strain cells remained alive. In case of BIOPOP-1, general viability of heat-adapted strain was higher than that of WT strain, but there was no significant difference in the values between WT and heat-adapted strain. However, in case of BIOPOP-2, the heat tolerance of heat-adapted strain increased substantially, and the results being significantly different compared to WT cells. In addition, an interesting observation was revealed that a strain with lower basal heat tolerance (BIOPOP-2) could extend its upper threshold by a greater value, while strain with higher basal heat tolerance (BIOPOP-1) would raise its upper limit to a very marginal extent. It might be considered that all bacteria have certain capacity to increase their stress tolerance limit. The lower the base values, the higher will the increment be, and higher based values mean there is less room for expansion.

Cross protection is based on mechanism that closely related responses are generated by different stress conditions [31]. In other words, different types of stresses lead to a common or similar type of response, as well as specific response by some stresses [17]. The strains in this study also expanded their cross-protection against multiple stress conditions such as high acidity, bile and salinity as a result of heat adaptation compared to WT strains. Probiotics must withstand multiple stress conditions to be able to colonize a colon of human in abundant numbers [38]. Before reaching the intestinal tract, probiotic bacteria must first survive acidic environment of the stomach generated by gastric juice [16]. In this experiment, heat-adapted strains exhibited higher level of acid tolerance than the control group. Upon reaching the intestine, probiotic bacteria face with another challenge, which is bile salts. It was confirmed that heat-adapted strains grew better than WT cells when they were exposed to 0.5% and 1% bile salts for 3 h. Lactic acid bacteria can also be exposed to osmotic pressure during manufacture processes when additives such as salt or sugar are added to the product. Osmotic changes in the environment could rapidly damage essential cell functions, and bacteria need to adapt to such a change in order to survive [8]. They were exposed to 20% NaCl for 2 h and 24 h, and heat-adapted strain again demonstrated higher level of stress tolerance than WT cells. Overall, the bacteria became more tolerate to the above mentioned stress conditions they might face during manufacturing and ingestion processes.

The analysis of fatty acid contents was carried out to determine the cause of increased heat tolerance. The fatty acid composition and the ability of the cells to tolerant the above mentioned stresses are closely related. Heat adaptation to high temperatures can change the chain length of the membrane fatty acid, which can be raised with increasing temperature, and the short-chain composition of the membrane fatty acid increases [39]. However, in our study of *S. thermophilus*, chain length does not appear to be the main regulating mechanism that reacts to changes in the temperature regime (Table 2) [40]. Compared to BIOPOP-1 and BIOPOP-2, the heat-adapted strains increased their heat tolerance, but the BOPOP-1 heat-adapted strains decreased their short chain content and the BIOPOP-2 heat-adapted strains have increased. Therefore, there was no correlation between heat adaptation and overall chain length (short chain length vs. long chain length). Unlike chain lengths, the relative composition of fatty acids seems to play an important role of heat adaptation. Many researches have shown that lowering concentration of unsaturated fatty acids (UFAs) or increasing concentration of saturated fatty acids (SFAs) is decreased membrane fluidity and related to higher heat tolerance [11, 23, 34]. In our study, the modifications in *S. thermophilus* membrane fatty acid composition were clearly linked to its heat tolerance enhancement induced by constant heat shock to cells [25]. The membrane adaptation of *S. thermophilus* cells as a response to being exposed to high temperatures was clearly indicate a decrease in UFA/SFA ratio [41]. It suggests that composition of the cellular fatty acids plays an important role in the response to heat stress in these strains. The SFA enhances acyl-chain packing in the membrane, and thus increases van der Waal interactions between the chains, which consequently leads to decreased membrane fluidity [42]. And this raises its ability to withstand multiple stresses. The amount of SFAs capable of increasing acyl-chain packing in the cell membrane is considered to be one of the most important factors for successful growth under various stress environments [4]. Our results were similar to those previously described other bacteria such as *Lactococcus lactis* [4], *Enterococcus faecium* [23] and *Pediococcus* spp. [43].

BIOPOP-1 and BIOPOP-2 strain were equally affected to decrease in C18:1 content. These changes are characterized by organisms using anaerobic pathway of fatty acid biosynthesis, in which the majority of the decrease in unsaturated fatty acids in unsaturated fatty acids is in C18:1 [44]. Thus, like *Avelino* et al. reported [11], the modifications of the cell membrane composition in response to heat stress serve the purpose of maintaining a degree of fluidity compatible with life. Also, heat-adapted strains only showed hexanoic acid (C6:0) by the heat adaptation. We predicted that a related increase in C6:0 level probably due to the fact that a decrease of the oleic acid (C18:1) was induced to produce hexanoic acid (C6:0) during heat adaptation period [25]. We confirmed that the BIOPOP-1 strain is naturally more heat tolerant than BIOPOP-2. Fatty acid analysis revealed that the SFA contents of the BIOPOP-1 strain was higher than BIOPOP-2, and specifically, behenic (docosanoic) acid (C22:0) was identified only in the BIOPOP-1 strain. Through these results, we reaffirmed that the SFA contents affects the heat tolerance, and the higher the saturated fatty acid content, the higher the heat tolerance.

In this study, it was discovered that heat tolerant *S. thermophilus* strains can be obtained through heat adaptation procedure. Heat tolerance of heat-adapted strains was higher than that of WT strains and exhibited higher tolerance to other stress conditions like acid, bile salts and salinity. The physiological description of these stress tolerance enhancement after heat adaptation is provided by the membrane fatty acid composition observed for *S. thermophilus* cells. Heat-adapted strains showed UFA/SFA ratio inversely proportional to adaptation in constantly increasing heat temperatures. New SFA composition was observed and consequently the increase of SFA composition causes to decrease of membrane fluidity in the bacterial membrane. It could also explain the increase in D-values found for *S. thermophilus* in response to the increase in heat temperature. However, the changes observed in the membrane fatty acid composition are not enough to explain the influence of the various stress tolerance enhancement of *S. thermophilus*. Thus, further research is required to determine the exact molecular mechanisms for heat adaptation. This method proved to be useful in the dairy industry, and can definitely be utilized in various industries.

## Supplemental Materials

Supplementary data for this paper are available on-line only at http://jmb.or.kr.

## Figures and Tables

**Table 1:** The results of cross protection against acid, bile salt and salinity condition.

			BIOPOP-1		BIOPOP-2	

			WT	Heat-Adapted	WT	Heat-Adapted
Acid	Con.^a^	log CFU/ml	9.21±0.06	9.21±0.07	9.12±0.1	9.23±0.03
	pH 2	log CFU/ml	4.54±0.04	5.80±0.01	4.43±0.07	6.95±0.01
		SR ^b^ (%)	0.002%	0.48%	0.002%	0.53%
	pH 3	log CFU/ml	9.10±0.05	9.14±0.01	8.97±0.08	9.19±0.003
		SR ^b^ (%)	78.18%	83.13%	69.81%	91.76%
Bile Salt	Con. ^a^	log CFU/ml	8.32±0.03	8.89±0.08	7.47±0.1	9.18±0.03
	0.5%	log CFU/ml	6.57±0.02	8.76±0.03	4.78±0.06	9.18±0.01
		SR ^b^ (%)	1.75%	71.82%	0.19%	98.91%
	1.0%	log CFU/ml	ND ^c^	4.36±0.06	ND ^c^	6.62±0.08
		SR ^b^ (%)	-	0.29%	-	0.28%
Salinity	Con. ^a^	log CFU/ml	9.20±0.02	8.96±0.08	8.21±0.09	8.66±0.04
	2h	log CFU/ml	8.24±0.01	8.65±0.004	6.95±0.03	8.13±0.01
		SR ^b^ (%)	10.88%	47.62%	5.35%	29.34%
	24h	log CFU/ml	6.67±0.01	7.53±0.05	5.42±0.03	7.56±0.01
		SR ^b^ (%)	0.29%	6.63%	0.16%	7.88%

The viabilities are expressed as mean±standard deviation of the measurements of three biological replicates.

^a^Con : Control, cells under no stress.

^b^SR : Survival Ratio (%)

^c^ND : Not Detected

**Table 2:** Comparison of relative fatty acid compositions between WT and Heat-Adapted strains by *Streptococcus thermophilus* BIOPOP-1 and BIOPOP-2.

Fatty acid (FA) composition	BIOPOP-1		BIOPOP-2	

	WT	Heat-Adapted	WT	Heat-Adapted
C6:0 (%)	ND ^a^	0.34±0.59	ND ^a^	0.35±0.61
C16:0 (%)	18.41±0.27	14.36±0.16	16.36±1.29	22.78±1.28
**Sum of Short chain FA**	**18.41±0.27**	**14.70±0.96**	**16.36±1.29**	**23.13±1.61**
C18:0 (%)	14.02±0.37	13.89±0.06	6.65±0.58	9.28±0.77
C18:1n9c (%)	18.7±0.30	14.99±0.55	25.97±1.32	21.61±0.29
C20:0 (%)	9.43±0.13	13.54±0.23	2.05±0.03	1.96±0.11
C20:1 (%)	36.9±0.49	39.25±1.55	47.25±2.68	43.01±2.04
C22:0 (%)	0.70±0.40	1.19±0.4	ND ^a^	ND ^a^
C22:1n9 (%)	2.31±0.09	3.24±0.02	1.71±0.45	1.01±0.05
**Sum of Long chain FA**	**81.59±0.16**	**85.30±0.56**	**83.64±0.74**	**76.87±0.93**
**Total**	**100**	**100**	**100**	**100**
**SFA ^b^ (%)**	**42.09±0.33**	**42.52±2.14**	**25.06±1.79**	**34.37±3.11**
**UFA ^c^ (%)**	**57.91±0.19**	**57.48±1.24**	**74.94±1.03**	**65.63±1.80**
**UFA/SFA ratio**	**1.38**	**1.35**	**2.99**	**1.91**

The viabilities are expressed as mean±standard deviation of the measurements of three biological replicates.

^a^ND : Not Detected

^b^SFA : Saturated Fatty Acid

^c^UFA : Unsaturated Fatty Acid
