# Supplementary material for: Changes in Cell Membrane Fatty Acid Composition of Streptococcus thermophilus in Response to Gradually Increasing Heat Temperature
Source: J Microbiol Biotechnol. 2020 Mar 2;30(5):739–48. doi: 10.4014/jmb.1912.12053 (PMC9745660; doi:10.4014/jmb.1912.12053)
Supplement: Supplementary file 1 [file JMB-30-5-739-supple.pdf]

J. Microbiol. Biotechnol.
https://doi.org/10.4014/jmb.1912.12053

Table S1 Taxonomic reports based on the 16S rRNA sequences of BIOPOP-1 and BIOPOP-2.

	Subject						Score		Identities	
	Accession	Description	Length	Start	End	Coverage	Bit	E-Value	Match/ Total	Pct.[a](%)
BIOPOP-1	NR_042778.1	*Streptococcus thermophilus*	1539	20	1493	95	2691	0.0	1471/ 1477	99.80
BIOPOP-2	NR_042778.1	*Streptococcus thermophilus*	1539	24	1501	96	2652	0.0	1475/ 1491	99.25

a Pct. : Percentage (%)
